# What's trust got to do with research: why not accountability?

**DOI:** 10.3389/frma.2023.1237742

**Published:** 2023-11-13

**Authors:** Moréniké Oluwátóyìn Foláyan, Bridget Haire

**Affiliations:** ^1^Department of Child Dental Health, Obafemi Awolowo University, lle-Ife, Nigeria; ^2^Kirby Institute, University of New South Wales, Sydney, NSW, Australia

**Keywords:** power, vulnerability, voluntariness, liability, exploitation

## Abstract

This paper explores the intricate dynamics of trust, power, and vulnerability in the relationship between researchers and study participants/communities in the field of bioethics. The power and knowledge imbalances between researchers and participants create a structural vulnerability for the latter. While trust-building is important between researchers and study participants/communities, the consenting process can be challenging, often burdening participants with power abrogation. Trust can be breached. The paper highlights the contractual nature of the research relationship and argues that trust alone cannot prevent exploitation as power imbalances and vulnerabilities persist. To protect participants, bioethics guidance documents promote accountability and ethical compliance. These documents uphold fairness in the researcher-participant relationship and safeguard the interests of socially vulnerable participants. The paper also highlights the role of shared decision-making and inclusive deliberation with diverse stakeholders and recommends that efforts should be made by researchers to clarify roles and responsibilities, while research regulatory agents should transform the research-participant relationship into a legal-based contract governed by accountability principles. While trust remains important, alternative mechanisms may be needed to ensure ethical research practices and protect the interests of participants and communities. Striking a balance between trust and accountability is crucial in this regard.

## Introduction

Bioethics is dedicated to identifying, studying, and addressing conflicts that arise from competing values or goals during the planning and execution of health-related life sciences research (Centre for Ethics and Humanities in the Life Sciences, 2020). Research ethics, an essential branch of bioethics, has its foundations in a history marked by instances of abuse and exploitation (Dhai, 2017). As a result, research ethics places a strong emphasis on preventing exploitation, encompassing the protection of study participants from exploitation and the safeguarding of the communities involved in supporting the research (Gbadegesin and Wendler, 2006; Bromwich and Millum, 2015).

In research involving human participants, there exist disparities in knowledge and power between the research team and the participants. Highly educated experts design research studies to address specific inquiries, utilizing data generated by the human participants. While researchers have a duty to ensure that participants provide informed consent and comprehend the use of their data, participants are not expected to possess a comprehensive understanding of all study aspects (O'Neill, 2003). These imbalances of power and knowledge contribute to the vulnerability of research participants, who must trust that researchers have effectively communicated relevant information and will act in their best interests in the event of unforeseen adverse events.

Trust is widely advocated as a fundamental value to be pursued in the planning and execution of collaborative research (Kerasidou, 2017). Researchers are encouraged to foster a trust relationship with their research participants and communities through active community engagement (McDavitt et al., 2016) and the informed consent process, which aims to ensure voluntary participation in research with an appropriate understanding of potential risks and benefits (Faden et al., 2005). In the field of bioethics, “trust” is typically understood as an attitude adopted by one party toward another, involving the acceptance of vulnerability in anticipation of goodwill (Geransar, 2016). This type of trust is generally applied within a specific domain of action, where one party entrusts another party with practices for which the latter holds personal or professional responsibility. Building trust, however, requires ongoing effort and long-term commitment (McDonald et al., 2008; Guillemin et al., 2016).

While trust has been extensively explored in interpersonal contexts, its examination in the researcher/participant relationship is less extensive. Interpersonally, trust-building implies that both parties enter into a relationship where one willingly exposes vulnerability to the other, who possesses influential power over the entrusted actions (Jones, 2012). It assumes that the trusted party holds goodwill toward the trusting party (Wright, 2010) and will go beyond basic obligations to protect them (Baier, 1986). Establishing a trust relationship necessitates the voluntary abrogation of power by the trusting party, based on the assumption that goodwill should prevent exploitation due to the trusted partner's moral agency (Kerasidou, 2017). In research, trust-building between researchers and the community is crucial for fostering collaborative and reciprocal relationships, and the informed consent process is considered a vital step in building trust. However, it often places the burden of power abrogation on the study participants or community, relying on the assumed goodwill of the researcher. The concept of trust also acknowledges the possibility of betrayal (Cooke, 2019).

In summary, trust entails one party in a relationship abrogating power, becoming vulnerable voluntarily on an assumption that good will should prevent any form of exploitation or betrayal because the trusted is a moral agent (Jones, 2012). In this paper, we examined the concept of power, vulnerability, voluntariness and goodwill in the context of a “trust” relationship between researchers and study participants/communities and the potential for exploiting this “trust” relationships create in research. We also addressed how the development of reliable measures of accountability provide critical scaffolding of the trust relationship between researchers and study participants/communities. Here, we define accountability as being answerable, or responsible to provide an account for: actions, conduct and the discharge of duties (Checkland et al., 2004).

## The ubiquitous expectation of study participants/communities to “trust” researchers

Trust as a sought goal in research/participant relationship places the onus of cultivating this relationship on the researcher (Christopher et al., 2008). It thereby assumes that researcher and research institutions have trustworthiness as a virtue on which to build a trusting relationship. The trustworthiness of an institution is inferred by its broad reputation, perceptions of its internal governance and ethical approval systems, and whether participants had some personal connection with the institution (Guillemin et al., 2018). When trying to build trust with a population with strong reasons to mistrust institutions and researchers, there is more emphasis on building the quality and depth of the relationship between an individual researcher and the study participants/community (Guillemin et al., 2018).

Trust makes use of the natural social tendencies of humans to simulate a kinship relationship. The kinship facilitates the process of quick forgiveness and or forgetfulness of momentary fear or resentment when faced with unpleasant experience imposed by the researcher. This possibility increases as the bond gets stronger (Cooke, 2019) thereby making study participants/communities vulnerable to exploitation. Without an acknowledgment of this vulnerability, there is an assumption that both parties have equal power, autonomy and access to health and social goods, which can be grounds for exploitation.

In the literature that discusses trust in the interpersonal context, the conceptual difference between “trust” and “reliance” is emphasized (Jones, 2012). Reliance connotes dependability but not necessarily dependability resulting from voluntary abrogation of power. It is a contractual relationship based on self-interest and consideration of vulnerabilities in the relationship (Baier, 1986). Trust, on the other hand, is characterized by a dependency upon the trustee to act as a moral agent according to the interests of the one who bestows trust, irrespective of whether those interests have been fully explicated, and despite other competing interests. Clearly, a research relationship is one based on reliance –a contracted relationship – that is muddled with elements that require study participants to trust researchers to act as moral agents who are obliged to respect the written and unwritten codes of the contractual relationship, due to the inherent uncertainties and risks involved in research. The requirement for “trust” in this contractual relationship is a non-legal based good will contractual relationship that is drawn between the researcher and the research participants. It is not “trust” as a value that is required to be elevated the level of a research principle nor should be elevated to that level knowing that such relationships – like all trust relationships and because of its social nature – are prone to betrayal (Gambetta, 1990).

Trusting involves much more morally and psychologically than mere reliance. There is less scrutiny of intent with the initial assumptions being that the action of the trusted is in good faith. There is an explicit belief in the trusted not to harm and to willingly protect. Trust is akin to giving the power for exploitation (McLeod, 2021) and to assume that trust will exclude exploitation in research-study participants/study community relationship is a failure to acknowledge the events of the past (Baier, 1986). It is therefore myopic to assume that a contractual relationship of trust between parties of unequal power and vulnerability will not be exploited (Baier, 1986). This assumption is flawed because it is based on a belief that the contractual relationship is a *cooperation between rationally calculating individuals, each seeking mutually advantageous outcomes* (Nussbaum, 2006). The assumption that relationship building between research-study participant/study communities is a voluntarily built relationship that can be willingly nurtured through trust, is a failure to acknowledge the affective element of trust building and relationship forming; that trust is a side effect of relationships with others, their behavior and one's familiarity with them (Nussbaum, 2006). The possibility for exploitation does not preclude the possibility for deep and meaningful relationships.

The recognition that the research relationship contains the possibility for exploitation of the vulnerabilities of study participants/communities is the reason for multiple guidance documents on bioethics (Permissible Medical Experiments, 1949; World Medical Association, 1964; National Commission for the Protection of Human Subjects of Biomedical and Behavioral Research, 1978; AVAC and UNAIDS, 2011; UNAIDS and WHO, 2011; WHO, 2011; International Ethical Guidelines for Health-Related Research Involving Humans, 2016; World Health Organization, 2016b; Aeras, 2017) that emphasize participants' protection. The documents recognize that research makes study participants/study communities vulnerable to researchers' choices; and this comes with an obligation for protecting study participants (Goodin, 1985). The guidance documents promote accountability of researchers to communities and funders and compliance with ethical norms. These documents are scaffoldings that make researchers accountable not just to the aims of their research, but to the broader goals of ethical research practice that respect the interests of participants, even in cases where interests might not have been pre-specified. This improves equity and fairness in the relationship between the two parties in the deployment of the obligations within the contractual relationship (Baier, 1986). Research guidance documents focus of the development of contractual agreement that promotes accountability and compliance. Research governance using these guidance documents imbue research institutions with the quality of “trustworthiness.” Researchers need to be held accountable to ethics guidelines that protect study participants/community; and promote accountability.

During clinical studies, when researchers collect data and specimens from individuals and communities, the study participants entrust their health and health related information to the researcher (Belsky and Richardson, 2004), and they take physical, economic, emotional, social and legal risks for the research (Lidz et al., 2004) howbeit these risks should have been minimized through ethics committee review (Emanuel et al., 2000). Study participants/communities pre-supposedly assume that their data and the specimens they provided will be handled in good faith as outlined in the informed consent (O'Neill, 2002), and that the researcher will hold true to the formal and informal relational contract drawn between the two parties (de Melo-Martín and Ho, 2008).

The recognition of this contractual relationship – one that acknowledges that the strong have a duty to look after the weak and the need to address moral claims of the vulnerable (Goodin, 1985) – obliges researchers to provide study participants' access to ancillary care (Lidz et al., 2004). The discussion on the limits and scope of ancillary care recognizes that a responsibility exists on the part of the researcher, and the responsibility is a contractual trade (exchanges occur) drawn on a moral obligation of a duty to care. The level of reliance or dependence of the researched on the researcher (Lidz et al., 2004) defines the scope and depth of this obligation. In non-clinical studies where researchers are not able to invest the time to build relationships – not necessarily out of a lack of interest in doing this but because the investment in a long-term process for a short-term outcome may not be viewed cost-effective — other paradigms of relationship building that are not based on trust are needed. Relationships built in non-clinical studies will need to operationalize the duty to care using a non-medical model that protects study participants from harm. These discussions acknowledge that the relationship is not one of bilateral equity — the researcher holds the power.

## The abrogation of power by study participants/communities during the conduct of research

The concept of “vulnerability” of study participants in the contractual relationship between researchers and study participants/communities connotes a sense of powerlessness. It assumes that there is potential for the participants communities to be harmed by research; conversely, study participants/communities may be subject to harm because they cannot provide voluntary consent to study participation, should the research be beneficial (Bracken-Roche et al., 2017). While entrusting oneself to the uncertain risks and benefits of research makes all study participants/communities vulnerable, those socially vulnerable due to poverty, stigma, or inability to defend themselves are more vulnerable than others, as they have *identifiably increased likelihood of incurring additional or greater wrong* (Hurst, 2008). Multiple studies have enunciated how poorer communities in the North-South research collaborations are more vulnerable because of their greater reliance on the *expectation, whether justified or not, of benefits associated with participation* (The National Health and Medical Research Council, the Australian Research Council and the Australian Vice-Chancellors' Committee, 2014); they are therefore easier to manipulate (The National Commission for the Protection of Human Subjects of Biomedical Behavioral and Research, 1979). This form of vulnerability is a relational one that equally needs to be safeguarded by measures independent of the researcher (Luna, 2009). In effect, the scale of power abrogation by study participants/communities who are socially vulnerable is much more than that abrogated by those who are not socially vulnerable. Researchers often define the depth and scope of the engagement they have with those being researched (O'Neill, 2002), and they also have the discretionary power handling the data and specimens shared with them. This imbalance in the power relationship further makes study participants/communities vulnerable to exploitation.

Abrogation of power by study participants/communities does not preclude their ability to exercise other powers, as the abrogation of power is not absolute (O'Neill, 2002). Multiple histories highlight how study participant/communities have exercised these powers often disrupting research and leading to loss of huge resources. The tenofovir pre-exposure prophylaxis research controversy – sometimes known as “the failed PrEP trials” – is one example. In this instance, local communities whose voices were initially unheard about the need to make modifications to study protocols *spoke up* by stopping further recruitment of study participants into the trials (Folayan and Peterson, 2020). Another example is that described by Kingori (2015a,b), who demonstrated how research participants in sub-Saharan Africa are presented with “empty choices” by ignoring significant structural and contextual factors in resource-limited settings that constrain access to health care, and how ignoring this constraint leads participants to take part in research in order to access health care services, while not taking the study products, thereby leading to research failures.

## Is trust precluded from researcher-study participants/community relationships

The goal for researcher-study participants/community relationship is the development of partnerships. Partnerships require that research activities respond to the needs of the host community and are designed and implemented jointly by researchers and community representatives. Partnership promotes the sharing of equitable, collaborative decision-making power between communities and researchers while recognizing and engaging the allegiances, power dynamics and vulnerabilities of diverse stakeholders engaged through inclusive deliberation. It is a time-consuming process that focuses on building competency and trust. It may be one of the building blocks for research relationships planned for the long term.

Trust in researchers and study participants/community relationships has its own nuances. Trust requires that the research team is responsive to the vulnerability of the study participants/communities, the limit of which needs to be defined jointly. Trust is, however, an emotive relationship of dependency associated with the risk for possible exploitation of the vulnerable. In trust-dependent relationships, exploitation may be subtle and take place over a long time without communities recognizing these signs of exploitation. Trust building is largely dependent on the personal characteristics of those who negotiated the relationship. When individuals who negotiated the terms of trust relationship between the two parties are no longer present, the unwritten terms of the dependency may be lost to others or members joining the team, thus opening avenues for exploitation. Trust cannot be enforced, and its magnitude does not correlate with the magnitude of expectations from both parties. For example, although the research participant/community only partially trusts the researcher, that does not imply that they partially contribute data and specimens to the research.

## Discussion

Efforts to ensure clarity in the roles and responsibilities in research relationships are evolving. One of these advances is that of ensuring clarity in the relationship between researcher and the research, with the aim of preventing implicit or explicit exploitation. One such possible effort is evolution of the moral-based contractual relationship drawn between researchers and study participants to a legal-based contractual relationship. Prior to now, Folayan et al. (2019) have argued for the evolution of research-participants' relationship into a labor contract governed by labor principles and enforceable by labor laws. Others have also argued in this direction (Winsberg et al., 2014). A labor relationship between research and research participants/communities will require the drawing up of explicit contracts that places both parties in a relationship governed by accountability principles and values. A “trust” element in the relationship will be required to strengthen the relationship rather than formally or informally governing the relationship.

Research regulation focuses on preventing exploitation and ensuring that researchers are accountable for the conduct of the research according to agreed terms and conditions. Where there is community distrust of research and researchers, this limits access to the possible opportunities accessible through research participation and may leave communities isolated. As social beings who need social interactions, trust-based relationships in research cannot be completely excluded. Trust is, however, an interpersonal factor that is essential in facilitating exchange relationships in which the researcher is perceived as having integrity, is willing to reduce research uncertainty, ensures confidentiality, has the expertise, and is tactful, sincere, congenial, and timely (Moorman et al., 1993). Trust is not a transactional resource and this is particularly important in a research relationship that is fraught with a history of unresolved distrust. Research and research processes also do not lend themselves to processes that are dependent on interpersonal relationships (Kristiansen and Bloch-Poulsen, 2011). It is therefore important that the research enterprise operates a system of engagement with communities and participants to which they can be held clearly accountable.

In view of this reality, for numerous reasons, research relationships need to exclude trust as a formal accounting tool for success. First, the agents involved in research have messy and conflicting interests and values, which make undue pressure for research regulators to cultivate and maintain trust in an otherwise contractual relationship. Second, trust is a subjective phenomenon, and it makes nonsense of efforts to define researcher obligations based on weights or measures of trust. Third, trust is either present or absent, and its absence does not preclude parties from being accountable. Being non-accountable breeches trust. There is, therefore, a need to define and monitor expected researcher-study participants/community relationships by objective measures that can be monitored.

Accountability, as an ethics operational principle, can enable research communities to hold researchers liable. It is a process that requires an accounting responsibility to some external authority, thereby encouraging adherence to agreed behaviors. This in turn promotes ethical behavior, as the norms and standards of behavior generated in response to the accountability mechanisms will include the protection of both the participants and their communities (Dubnick, 2003). In addition, accountability breeds more interdependent behaviors, and both parties experience greater satisfaction (Fandt, 1991).

Researchers may hold themselves accountable because of their allegiance to a social status and membership of the research community that has high social respect, and because it is in their interest to be perceived as credible by external assessors (Miller and Weijer, 2006; Tamin, 2010). Researchers often assume less of this role in the accountability structure because of their own narratives of their relationship with the community; thus, they are better able to recount events to the external audience that often exonerate them of blame by assuming praiseworthiness because of their pursuit of good of the public (Frederickson and Hart, 1985).

We propose that in research-participants relationships, accountability-liability be pursued wherein actions are guided by rules that carry sanctions for non-compliance (Shklar, 1986). Reward for compliance is measured by study participants' recruitment and retention, while the legal court of law can sanction for violations of contractual agreement. Folayan and Allman (2011) have argued for this kind of contractual relationship between researchers and research communities with the rules governing the relationship mediated by labor laws. Folayan and Peterson (2020) expanded on this concept of contractual accountability relationships between researchers and research communities and argues for the formalization of a wage-pay model relationship that makes for a shift from dependency on ethical guidelines which rarely has the bite of the law and has been subject to abuse and continued exploitation of research communities (Benatar, 2000). Positive interpersonal interactions are still essential for functioning of an accountability mechanism that is dependent on liability norms for its operations (Nxumalo et al., 2018).

An ethics guidance document that recognizes this principle of accountability is the *Good Participatory Practice Guidelines Governing Biomedical HIV Prevention Trials* (AVAC and UNAIDS, 2011). However, it promotes an accountability-answerability model that depends on a trust and respect relationship. For this reason, the guidelines lack a process of implementing this principle, as much is left to researchers and communities to make the best of the relationship. This limited interpretation of accountability is reflected in the definition of accountability in the *Good Participatory Practice Guidelines for TB Drug Trials* (Critical Paths to TB Drug Regimen, 2012) and the *Good Participatory Practice Guidelines for Trials of Emerging (and e-emerging) Pathogens* (World Health Organization, 2016a), both of which were adapted from the *Good Participatory Practice Guidelines for Biomedical HIV Prevention Trials*. Though these documents promote efforts to recognize the expertise and “voice” of study participants/communities, this acknowledgment is not synonymous with equity in power; neither is it the prerogative of the researcher only to build trust, knowing that it is earned and cannot be demanded.

An approach centered on accountability in participatory practice entails outlining the essential procedures for research teams to establish a transparent and responsible rapport with community collaborators. These steps should also be clearly documented in the pertinent normative materials referenced during the ethical review process. Research teams should proactively address each step within their ethics application, and community partners should receive comprehensive documentation of the expected procedures. This way, if the research team fails to adhere to the prescribed processes, community partners will possess evidence to file a formal complaint with the Institutional Review Board. Furthermore, research ethics training for research teams should encompass instruction in participatory research practices and stress the significance of being accountable for ensuring that processes are executed in accordance with the research protocol.

However, we recognize the limitations inherent in this discussion. While we advocate for a new approach to the interaction between researchers and study participants/communities, one grounded in accountability frameworks designed to rectify the power imbalances, we are mindful of the subtleties that these frameworks may not encompass. These accountability frameworks may not fully address the processes required to build trust within the research team and between research teams and the host institutions. They may also not sufficiently address the capabilities of ethics committees in mitigating the risks of exploiting vulnerable communities.

It is also important to acknowledge that the potential for exploitation is an inherent concern in the design and execution of clinical trials. Nevertheless, we acknowledge that the model of engagement based on accountability is applicable to a broader range of research contexts beyond clinical trials.

## Conclusion

Going forward, the issue of trust and trust building in the researcher-community relationship needs to be interrogated further. Relationships built primarily on trust can be exploited and, sadly, are difficult to judge in formal settings. Trust is essential in team relationship building, and it is an essential ingredient for interpersonal interactions, even within relationship models built on accountability. For researcher-community relationships that have been fraught with distrust and exploitation for decades, it is important that other models of engagement, which shift from a trust-based model to one that fosters accountability-liability, be explored. This is more important in the evolving world of “new normal” practices in research, where the norm is fast-tracked research, especially during epidemics and pandemics, and trust building will often be dismissed. Unless accountability-liability models become the core operational model, the risk of exploitation and abuse becomes inevitable in the world of the “new normal” research practice.

## Data availability statement

The original contributions presented in the study are included in the article/supplementary material, further inquiries can be directed to the corresponding author.

## Author contributions

MF: Conceptualized the manuscript, developed the first draft, collated edits, and agreed to the final submission. BH: contributed extensively to the development of the manuscript and agreed to the final submission. All authors contributed to the article and approved the submitted version.
